# Improved Artificial Teeth

**Published:** 1888-01

**Authors:** D. Genese

**Affiliations:** Baltimore.


ARTICLE III.

### IMPROVED ARTIFICIAL TEETH.

BY DR. D. GENESE, BALTIMORE.

In dental prosthetics, one of the greatest annoyances met with is fracture of the porcelain in one or more teeth of a denture after the finishing. It may occur before it leaves the laboratory, when it can be rectified to some extent, thereby saving the patient the annoyance of a broken tooth soon after having a case made; but the trouble still remains the same to the operator or assistant. How much more so, when the patient leaves us to take a journey, fully satisfied that one thing at least is all right, when, lamentable to relate, just on the start a sudden closure of two over-arching teeth, one of which having received a flaw, gives way. What annoyance to all concerned—dentist, assistant and patient! Does the piece of work ever look the same, or is the patient satisfied? A dread is constantly present that the accident may occur again. Our client may charge us with carelessness, while we think the fault is on his side.

What is the cause of this trouble? It occurs in all forms of teeth, from the furnaces of every manufacturer, both in this country and abroad. Why? Because they place a lever in every tooth and use the pins as the fulcrum. They use materials having no affinity with each other, each one having to support the other by the bulk of material increasing at the parts uniting, not by fusion, but contraction, on each other, breaking up the unity of both tooth and base, trusting to the rivet-head of two small pieces of wire to hold all together.

And now comes the question, what causes the rivet-heads or pins to pull out from the porcelain instead of from the softer companion rubber, or from the backings in plate-work, instead of the tooth, in such cases?

In the first place, the tooth substance forms a hole or holes similar to a draw-plate, and the continued traction will pull the pins out as clean as from a draw-plate of steel; and the remark is often made, why don't they put heads to the pins? If we examine a tooth from which pins have drawn out, we will find it countersunk, with a clean, vitrified circular hole from the head to the surface.

Another consideration must be taken into account. Metal wire on tension will stretch, and the space left between the tooth and backing, or tooth and rubber, will, by capillary attraction, get filled with moisture and act as a hydraulic force, independent of the pressure of mastication.

We must also remember that in nearly all our work we depend upon teeth attached only by pins inserted in the centre, or nearly so, of the tooth or teeth having a constant leverage from both ends. Is it any wonder that such a frail substance breaks when we put the greatest strain on the weakest point?

It is not generally known how double-headed pins are inserted into the teeth, what pressure is put upon them during the process, or what has been done to weaken them previous to their getting into the hands of the dentist. First, the wire is drawn, then cut and headed; next, they are inserted into the body of the tooth by pressure, and when the teeth are biscuited, defects around them are filled in. Now comes the firing, and in this process they are twisted considerably out of shape. It would not do to send them out in this state, so poor pins are twisted again to look nice, and we have a faulty article to start with. Finally, the very insertion of the pins so weakens the tooth that the wonder is, how they stand so well.

How are we to overcome this trouble? For vulcanite work, do away with metal altogether; it leaves the porcelain solid throughout, and the contraction of the rubber, instead of leaving a space between it and the teeth, draws them (the teeth) closer to it and prevents the accumulation of debris, so detrimental to the dental piece and the health of the patient.

But you will say, what shall we do in metal work? Well, gentlemen, do the same, with a difference. Keep your porcelain solid throughout; make your metal cylindrical, from the cutting edge of the tooth to the root; at the same time, so arrange the metal that it permits of expansion and contraction of both porcelain and metal independently of each other.

I have brought specimens of both forms of teeth herein described, and would call your attention to the tube teeth of Ash & Sons, which resemble in some respects what I would show you. The difference is plainly perceived, both for the vulcanite and the metal work.

In the first mentioned I take advantage of a tripod groove running through the buccal and labial surface of the teeth, giving the greatest support where most needed, without weakening the grinding surface or the hold of the rubber to the teeth.

In the teeth for metal you will find the cylinder placed in the centre, a spring space running to the back receiving the greatest pressure in masticating, at the same time uniting the tooth to the plate by a large central holdfast, besides the ordinary backing which has already been burnt to the tooth, and will not, therefore, shrink or expand again like the ordinary backings riveted to pin teeth.

The combined strength and simplicity of arrangement,

I trust, will remove a great source of annoyance in our daily practice, besides giving greater satisfaction to our patients and substantial security to our work.—*Southern Dental Journal.*

